# Maternal Parity Effect on Spine Posture Changes and Back Pain During Pregnancy

**DOI:** 10.3390/healthcare12222202

**Published:** 2024-11-05

**Authors:** Michał Popajewski, Magdalena Zawadka, Alicja Wójcik-Załuska, Paweł Milart

**Affiliations:** 1 Department of Clinical Physiotherapy, Faculty of Health Sciences, Medical University, 20-093 Lublin, Poland; michal.popajewski@umlub.pl (M.P.); alicja.wojcik-zaluska@umlub.pl (A.W.-Z.); 2 Department of Sports Medicine, Faculty of Health Sciences, Medical University, 20-093 Lublin, Poland; 3 3rd Department of Gynecology, Medical University, 20-093 Lublin, Poland; pawel.milart@umlub.pl

**Keywords:** posture, spine, pregnancy, body mass index

## Abstract

**Background:** Pregnancy can significantly alter posture and stability, thereby affecting spine curvatures. A positive relationship between the number of full-term pregnancies and the prevalence of low back pain (LBP) has been reported previously. This study aimed to analyze the impact of pregnancy on spine posture and LBP. **Methods:** Thirty pregnant females who were nulliparous (Group 1, n = 15) or had one or two pregnancies (Group 2, n = 15) were examined using the photogrammetric method in the first, second, and third trimesters of pregnancy. Further, a correlation analysis was conducted among the body mass index (BMI), pain intensity (VAS scale), and spine posture parameters. **Results:** The parous groups did not differ significantly in the parameters of the spinal posture. The thoracic angle decreased in trimester II compared to trimester I (157.77° vs. 160.55°, *p* = 0.004), which, according to the measurement methodology used, means that the thoracic kyphosis curvature increased. BMI was associated with the angle of trunk inclination in trimester I in Group 1 (*r* = 0.54, *p* = 0.04), as well as with the thoracic angle in trimesters II and III in Group 2 (*r* = 0.54–0.62, *p* < 0.05). A statistically significant correlation between pain intensity and spine posture parameters was more frequently observed in Group 2. **Conclusions:** Parity does not affect spine posture during pregnancy or pain intensity. The intensity of LBP was associated with spine posture changes during pregnancy, but the character of association differs between groups of parity. Alterations in spine posture should be monitored during pregnancy to prevent back pain.

## 1. Introduction

The spatial orientation of the lumbar spine determines the setting of the central axis of the skeleton. This is related to the position of the pelvis. For this reason, the position of the sacrum or the entire pelvis may affect the shock-absorbing capacity of the entire spine and thus predispose the patient to the development of overload changes. Any change in the pelvic inclination angle may affect the depth of the curvature of the spine, leading to tissue overuse and back pain [1,2,3].

Body posture during pregnancy is associated with a shift of the center of gravity and center of pressure, which are the main indicators of posture stability. The lordotic angle increases when comparing pregnant women to non-pregnant women. The increased path length of the center of pressure observed during pregnancy suggests reduced stability [4,5,6]. Changes in body posture were observed during pregnancy and post-partum in standing and sitting positions [7].

Back pain is a common symptom in pregnant women but is often underestimated and undertreated [8]. Pain in pregnant women is mainly linked with myofascial dysfunctions [9]. Shifting the center of gravity forward during pregnancy increases the anterior tilt of the pelvis and thus deepens the curvature at the level of the lumbar spine. This forced posture increases the tension of the paraspinal muscles and contracture of the hip flexor muscles [2]. Spinal and pelvic pain affects more than half of pregnant women. In terms of back pain, 70% of the females experienced pain during their pregnancy. Factors associated with low back pain (LBP) included income status, frequent urinary tract infections, trimester, gestational weight gain, a history of LBP, and bad posture [10,11]. However, the relationship between the back pain experienced during pregnancy and posture remains unclear. Non-invasive methods of posture and spine evaluation help to understand this relationship. Surface topography (raster stereography) systems and photogrammetric methods capture digital photos to assess the pinpoint surface asymmetry and identify body landmarks [12,13]. Those methods allow for monitoring changes in posture during pregnancy.

There are evident effects on the biomechanics of women due to pregnancy, although the results of most parameters often contradict each other. Clinical experience suggests increased angles of lordosis and kyphosis in the spine as the most frequently observed posture alternations [1]. LBP is considered to be associated with an increase in lumbar lordosis due to hormonal changes in pregnancy and mechanical factors [14]. Thus, a greater number of pregnancies seems to contribute to a greater risk of musculoskeletal pain. Another factor contributing to LBP in the general population is age [15]. However, there is no agreement about the association between age and LBP during pregnancy [10,11]. LBP can be more frequent in women who gain more weight during their pregnancies. The increase in weight during pregnancy puts more pressure on the lower back, pelvic joints, and lower extremities, which can lead to a higher risk of experiencing LBP [10,11,16].

Females claim that back pain prevents them from working and affects their daily routines, including housework. LBP is a significant distressing factor for pregnant women and a public health problem [17]. For this reason, research focusing on the identification of posture’s role in the development of back pain is crucial.

The purpose of this study is to compare spinal posture parameters in the sagittal plane between pregnant females who have not given birth previously (nulliparous) and those who have given birth one or two times. Additionally, this study aimed to analyze the relationship among posture parameters, body mass index (BMI), and LBP in females during the first, second, and third trimesters of pregnancy. We have hypothesized that maternal parity affects posture and pain experience, and there is an association among posture, back pain, and BMI.

## 2. Materials and Methods

### 2.1. Subjects

The research was conducted on a group of 30 women who were examined three times in the first, second, and third trimesters of pregnancy. The attending physician’s consent and a note on the normal pregnancy progression were required to participate in this study. An a priori sample size estimation was conducted using G*Power 3.1 [18]. The calculations indicated that a sample size of 28 participants (14 per group) would be sufficient to notice a significant within-factors effect of the ANOVA with repeated measures. The sample size calculation was determined using the following parameters: an *α* value of 0.05, a power value of 0.80, 2 groups, 3 repeated measurements, and an estimated *ηp*^2^ value of 0.06 (moderate effect f = 0.25) [19]. Participants were divided into two groups: nulliparous (Group 1, n = 15) and one (n = 8) or two (n = 7) pregnancies (Group 2, n = 15, in total). Multiparous patients were enrolled with parity in terms of the number of deliveries (with 1 previous pregnancy or 2 pregnancies). Females who did not consent to participate in this study were not included. Informed consent and the study protocol were approved by the bioethics committee (KE-0254/196/2016). This study was conducted following the Declaration of Helsinki.

### 2.2. Procedures

**Figure 1:**
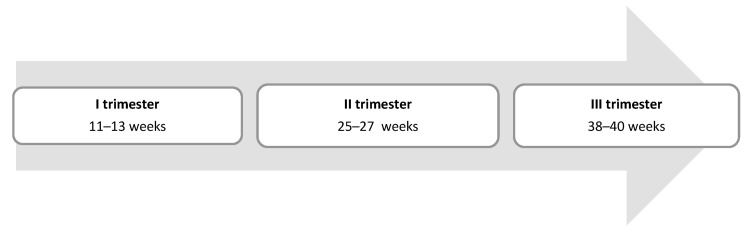
Observation timeline.

We conducted a physiological single-center observational study using screening data from pregnant women who underwent routine check-ups at the Gynecology Clinic, Medical University of Lublin, between July 2017 and December 2019. Participants voluntarily took part in this study. Spine posture, LBP intensity, and body weight were examined in each trimester of pregnancy. The observation timeline is presented in Figure 1.

**Figure 2:**
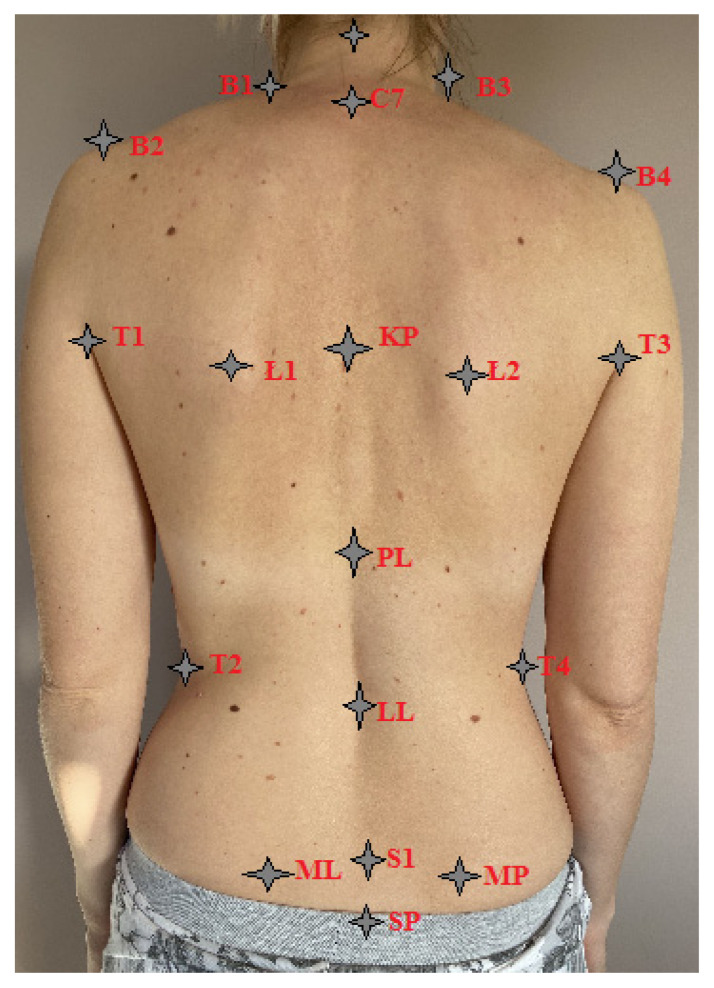
Woman’s back with markings of topographic points corresponding to the indicators in the topographic study.

The spine curvatures were examined using the MORA 4 Generation device [20] for the non-invasive diagnosis of postural disorders. The photogrammetric method is based on the projection of the moiré method. The examination of the patient was preceded by marking characteristic points on her body, which were visible after taking a photo. Cursors were placed at these points based on which parameters were being calculated (Figure 2).

Characteristic points were marked on the patient’s back, i.e., the occipital protuberance, the spinous processes of the vertebrae, the lower angles of the shoulder blades, and the posterior superior iliac spine. The steps of posture recording preparation in order are as follows:

1. Determination of shoulder height and waistline;
2. The patient stood with her back to the equipment at the examination distance (2.6 m);
3. Turned off all light sources in the room;
4. Started the camera and installed the program on the computer;
5. Recorded subsequent photos of the patient [21].

The parameter designation is as follows:

- C7 spinous process of the seventh cervical vertebra;
- KP measure of thoracic kyphosis;
- PL thoracolumbar transition;
- LL center of lumbar lordosis;
- S1 spinous process of the first sacral vertebra;
- L1 inferior angle of the left scapula;
- L2 inferior angle of the right scapula;
- ML left posterior superior iliac spine;
- MP right posterior superior iliac spine;
- T1, top of the left posterior axillary fold;
- T2 waistline on the left side;
- T3, top of the right posterior axillary fold;
- T4 waistline on the right side;
- B1 point connecting the shoulder line with the neck on the left side;
- B2 acromion process of the left scapula;
- B3 the point connecting the shoulder line with the neck on the right side;
- B4 acromion process of the right scapula;
- SP—the highest point of the gluteal cleft.

The parameters analyzed in the sagittal plane are as follows:

- Angle of inclination of the trunk. The inclination of the C7-S1 line in the sagittal plane was determined. The value of inclination is expressed in degrees. The value is positive if C7 is closer to the camera than S1.
- Depth of lumbar lordosis (GLL):

GLL = depth LL − PL(1)

- Lumbar angle (KLL):

KLL = 180 − (ALFA + BETA)(2)

- Depth of thoracic kyphosis (GKP):

GKP = depth KP − PL(3)

- Thoracic angle (KKP) [22]:

KKP = 180 − (BETA + GAMMA)(4)

ALPHA—inclination of the lumbar-sacral section.
BETA—inclination of the thoracolumbar section.
GAMMA—inclination of the upper thoracic spine.

**Figure 3:**
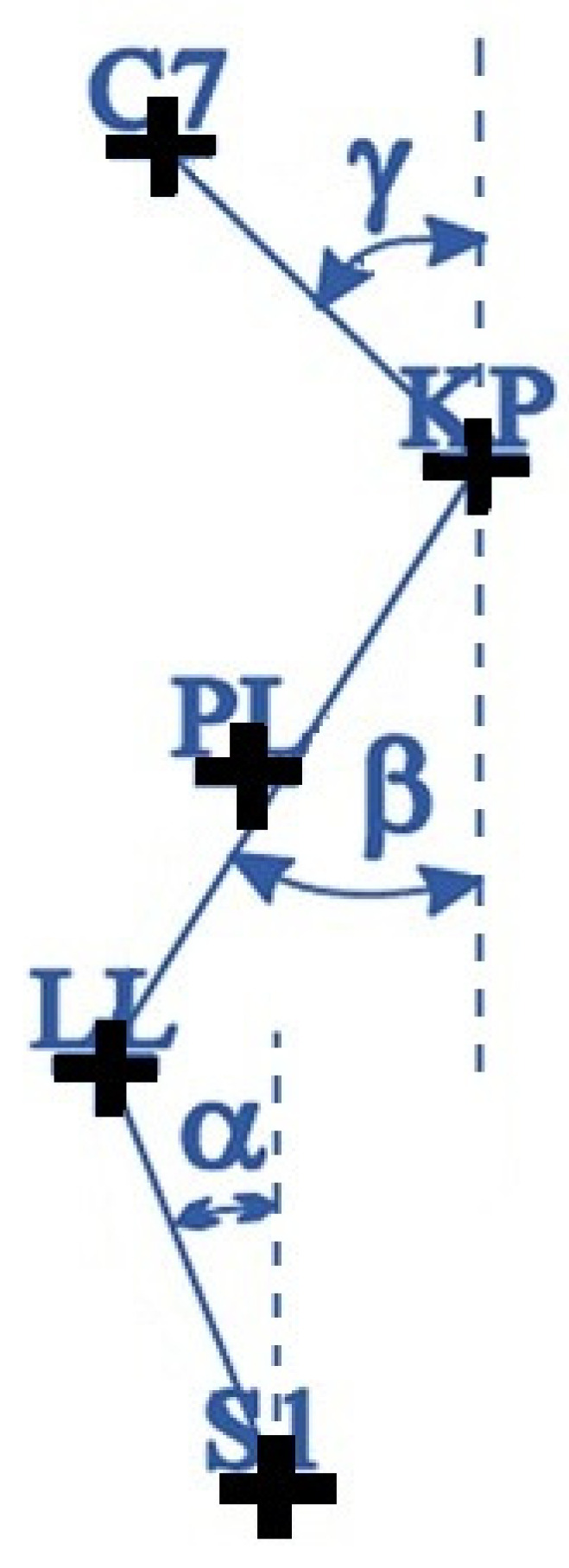
Determination of spine parameters. Characteristic points on the body were indicated with the symbol “+”.

The analyzed parameters are shown in Figure 3.

A greater thoracic and lumbar angle value means that thoracic kyphosis and lumbar lordosis decrease [23]. LBP was assessed using a 10-point analog visual VAS scale. Body mass index (BMI, [kg/m^2^]) was calculated by dividing body mass by body height squared. All measurements were taken by the same investigator (first author) and repeated three times (trimesters I, II, and III of pregnancy).

### 2.3. Data Analysis

Conformity of the distributions of the examined variables with a normal distribution was verified using the Shapiro–Wilk. Age, weight, height, and BMI were compared between groups using t-tests for independent groups. One-way ANOVA with repeated measures was used to calculate the effect of the parity groups (2) and pregnancy trimester (3) on spinal posture parameters. The significant results were analyzed further using the post hoc Bonferroni test. The partial eta square (*ηp*^2^) was used for effect size assessment; 0.01 is indicative of a small effect size, 0.06 is indicative of a medium effect size, and 0.14 is indicative of a large effect size. Pearson’s coefficient (r) was used to calculate the correlations among the parameters. The magnitude of correlation was assessed with the following thresholds: <0.1, trivial; <0.1–0.3, small; <0.3–0.5, moderate; <0.5–0.7, large; <0.7–0.9, very large; and <0.9–1.0, almost perfect [19]. A significance level of *p* < 0.05 was adopted, indicating the existence of statistically significant differences or relationships. Descriptive statistics included mean and standard deviation (SD). The database and statistical tests were carried out using STATISTICA (Version 14.0.0.15, TIBCO Software Inc, Palo Alto, CA, USA; 2020).

## 3. Results

There were no statistically significant differences between groups in terms of weight, height, and BMI. The females in Group 2 were slightly older than the females in Group 1 (32.80 ± 3.23 vs. 28.47 ± 4.39, *p* < 0.01). Details of comparisons are presented in Table 1.

ANOVA showed a statistically significant effect of the trimester on the thoracic angle and LBP experience (VAS). A post hoc analysis indicates that the thoracic angle decreased in trimester II compared to trimester I (157.77° vs. 160.55°, *p* = 0.004, both groups combined), which, according to the measurement methodology used, means that the thoracic kyphosis curvature increases. There was no statistically significant difference between trimester III and I (*p* = 0.17) and between trimester II and III (*p* = 0.48). VAS statistically significantly increases during pregnancy (trimester I: 1.6, trimester II: 3.23, trimester III: 4.47, *p* < 0.001, both groups combined). There were no statistically significant interactions between factors. Detailed results are shown in Table 2.

There were statistically significant correlations among BMI, age, and spine posture parameters in Group 2. Lumbar angle strongly and positively correlated with age in all trimesters (r = 0.64–0.79, *p* < 0.05). Thoracic angle in trimester II positively correlated with BMI in all trimesters (r = 0.58–0.62, *p* < 0.05). Thoracic angle in trimester III was statistically significantly correlated with BMI I; however, correlations with BMI II and III were very close to the statistical significance level (r = 0.51, *p* = 0.05). There were no statistically significant correlations in Group 1, except a negative relationship between trunk inclination and BMI in trimester I (r = −0.54, *p* = 0.04). A matrix correlation is presented in Table 3.

There was no statistically significant correlation among BMI, age, and VAS in either group. In Group 1, thoracic angles II and III were strongly and negatively correlated with VAS II and VAS III (r = −0.57–−0.72, *p* < 0.05). In Group 2, VAS II was statistically significantly associated with trunk inclination, thoracic depth, lumbar depth, and lumbar angle in trimester I, trunk inclination, lumbar, and thoracic depth in trimester II, and trunk inclination, thoracic depth, lumbar depth, and lumbar angle in trimester III. Moreover, VAS in trimester III was strongly and negatively correlated with lumbar angles II and III (r = −0.59–−0.64, *p* < 0.05). The results are presented in Table 4.

## 4. Discussion

This study aimed to compare spinal posture parameters in the sagittal plane between pregnant females who have not given birth previously (nulliparous) and those who have given birth once or twice. Additionally, the relationship among posture parameters, BMI, and back pain in females during the first, second, and third trimesters of pregnancy was investigated. The main finding is that parity did not affect the spinal posture or back pain parameters. The thoracic kyphosis curvature increases between trimesters II and I of pregnancy. The relationship among age, BMI, back pain, and body posture differs among parity groups. Those findings partially support the initial hypothesis.

The number of births is not a factor affecting spine posture. Previous studies determined how gravidity contributes to the development of lower back pain and lumbar sagittal balance. Güngör and Karakuzu reported that females with fewer than five pregnancies have less back pain and disability compared to those with five or more pregnancies. However, gravidity did not influence the sagittal balance parameters like lumbar lordosis, sacral slope, pelvic incidence, and pelvic tilt [24]. In the current study, age differences between parity groups may have a greater influence on spine posture than parity itself.

The main change in spinal posture during pregnancy was an increase in thoracic kyphosis between trimesters I and II. Surprisingly, no statistically significant differences were found in lumbar lordosis. We have found that during pregnancy, the experience of LBP gradually increases. Our findings are consistent with reports indicating a significant relationship between LBP and gestational age [10]. The current findings on spine posture align with previous research by Betsch et. al., who found a significant increase in thoracic kyphosis during pregnancy but no increase in lumbar lordosis [25]. Biviá-Roig et al. also reported no alterations in lumbopelvic position in pregnant females compared to nulliparous and postpartum women. However, there was an increase in the muscle activity of the trunk extensors. These results indicate that the extensor muscles of the trunk show adaptive responses to the increase in anterior loads during pregnancy [26]. An increase in body mass and the shift of the center of gravity exert additional static and dynamic loads on the axial skeleton, leading to an increase in thoracic kyphosis [2].

Another finding of the current study is a positive correlation between lumbar angle and age in Group 2. A greater lumbar angle means lower lumbar lordosis with age. These observations do not necessarily have to be related to pregnancy. The intervertebral disc flattens with progressing age, reducing the lumbar curvature; thus, reduced lumbar lordosis is normally expected [27]. However, a difference of approximately four years between the groups is probably not sufficient to significantly affect lumbar lordosis. It is also probable that the onset of the reduction in lumbar lordosis occurs after the age of 30; therefore, the correlation occurs only in Group 2. The impact of parity on lumbar lordosis needs further research since some previous studies have shown that pregnant females may experience a decrease in spinal curvature, as well as a reduction in the angles of lumbar lordosis and sacral inclination [28].

There was an association between thoracic angle in trimesters II and III and BMI in Group 2. This positive correlation means that more flattened thoracic kyphosis is associated with a greater BMI. Previous studies have not confirmed the relationship between back pain and body weight or the number of pregnancies [29]. However, the current study shows that the relationship between body posture and BMI differs between parity groups, but there was no statistically significant correlation among BMI, age, and VAS in either group.

The intensity of the experienced LBP (VAS) was related to thoracic angles in trimesters II and III in Group 1. This negative association means that increases in thoracic kyphosis are related to greater back pain. In Group 2, back pain in trimester I was associated with trunk inclination, thoracic depth, lumbar depth, and lumbar angle but not thoracic angle. The relationship between pain and lumbar angle means that greater lumbar lordosis is related to greater back pain in Group 2. The groups differed in terms of factors and the number of factors associated with LBP. A greater number of postural parameters associated with LBP was observed in Group 2 compared to Group 1, especially in trimester II.

The main limitation of this study is the lack of a control group of non-pregnant women with low back pain. This study aimed to focus on changes occurring during pregnancy and differences between groups experiencing LBP during pregnancy. It may also be interesting to compare these findings to non-pregnant women who also experience LBP. Future studies could include a follow-up with the same patients analyzed at 1 and 3 months postpartum or even compare data before pregnancy. Another important issue is the small sample size. It would be recommended to analyze a larger sample in future studies. However, to the authors’ knowledge, this is the first study comparing the body posture of a pregnant woman in terms of parity. The current study explains discrepancies in previous research on body posture during pregnancy and their relationship to back pain. A previous investigation reported that only 25% of prenatal care providers recommended treatment for back pain [29]. This confirms that back pain during pregnancy is underestimated and undertreated and needs further research for better understanding. The monitoring of posture misalignments and their relationship with back pain by physiotherapists may be useful for in-patient education and contribute to improving the quality of life of pregnant women by selecting appropriate forms of therapy.

## 5. Conclusions

Spinal posture during pregnancy was not affected by parity. In both groups, an increase in thoracic kyphosis and LBP was observed during pregnancy. In a group of females who have given birth one or two times, lumbar lordosis and thoracic kyphosis were associated with age and BMI, respectively. The intensity of LBP was strongly associated with spine posture changes during pregnancy, but the character of association differs between groups of parity. In nulliparous women, back pain was associated with increased thoracic kyphosis, while in women who have given birth once or twice, back pain was related to an increase in lumbar lordosis. Alterations in spine posture should be monitored during pregnancy to prevent back pain and select optimal treatment and education. The influence of parity on factors associated with LBP needs in-depth analysis in future studies.

## Figures and Tables

**Table 1:** Groups comparisons. Group 1 = nulliparous; Group 2 = multiple deliveries.

Variable	Group 1 (n = 15)		Group 2 (n = 15)		Statistics	
	Mean	SD	Mean	SD	t-Value	*p*
Age [years]	28.47	4.39	32.80	3.23	−3.08	<0.01 *
Weight [kg] I	64.80	15.02	61.07	9.43	0.82	0.42
Weight gain [kg] I–II	5.87	2.13	6.33	2.47	−0.55	0.58
Weight [kg] II	70.67	16.59	67.40	10.71	0.64	0.53
Weight gain [kg] II–III	13.47	3.56	13.20	3.21	0.22	0.83
Weight [kg] III	78.27	17.33	74.27	11.04	0.75	0.46
Height [cm]	166.00	7.16	165.27	4.59	0.33	0.74
BMI 1 [kg/m^2^]	23.42	4.58	22.31	2.95	0.79	0.44
BMI 2 [kg/m^2^]	25.54	5.04	24.63	3.38	0.58	0.57
BMI 3 [kg/m^2^]	28.27	5.05	27.15	3.51	0.71	0.48

* Statistically significant difference (*p* < 0.05).

**Table 2:** Results of ANOVA.

Variable	Trimester	Group 1		Group 2		ANOVA					
		Mean	SD	Mean	SD	Group F	*p*	Trimester F	*p*	Interaction F	*p*
Inclination [°]	I	1.13	4.05	3.07	3.67	1.28	0.27	0.41	0.67	0.81	0.45
	II	1.33	3.53	2.46	3.17						
	III	1.78	2.98	2.74	3.49						
Depth of thoracic kyphosis [mm]	I	22.87	9.69	26.80	10.23	1.12	0.30	0.83	0.44	0.24	0.79
	II	21.93	9.28	25.40	8.81						
	III	22.33	7.88	24.80	9.09						
Thoracic angle [°]	I	160.31	6.08	160.79	5.89	0.15	0.70	5.70	0.006 * η^2^ = 0.17	0.14	0.87
	II	157.15	5.69	158.38	5.64						
	III	158.71	5.95	159.18	4.98						
Depth of lumbar lordosis [mm]	I	−23.67	8.93	−27.60	10.50	1.59	0.22	0.40	0.67	0.03	0.97
	II	−23.00	9.41	−26.53	8.14						
	III	−23.00	7.70	−26.93	8.11						
Lumbar angle [°]	I	160.89	6.89	160.95	5.73	0.04	0.84	1.28	0.29	0.23	0.79
	II	159.59	5.48	159.67	7.02						
	III	159.91	4.83	160.94	4.44						
VAS	I	1.13	0.92	2.07	0.80	2.98	0.10	70.93	<0.001 * η^2^ = 0.72	1.69	0.19
	II	3.20	1.21	3.27	1.22						
	III	4.13	1.60	4.80	1.08						

* Statistically significant effect (*p* < 0.05).

**Table 3:** Results of Pearson correlations of posture parameters, age, and BMI.

Posture Parameter	Group 1								Group 2							
	Age		BMI I		BMI II		BMI III		Age		BMI I		BMI II		BMI III	
	r	*p*	r	*p*	r	*p*	r	*p*	r	*p*	r	*p*	r	*p*	*r*	*p*
Inclination [°] I	−0.24	0.39	−0.54	0.04 *	−0.51	0.05	−0.49	0.06	−0.09	0.74	−0.31	0.26	−0.30	0.28	−0.22	0.42
Depth of thoracic kyphosis [mm] I	−0.26	0.34	−0.41	0.13	−0.38	0.16	−0.41	0.13	−0.30	0.27	−0.32	0.24	−0.31	0.26	−0.27	0.33
Thoracic angle [°] I	0.06	0.84	−0.19	0.49	−0.19	0.49	−0.11	0.69	0.26	0.34	0.05	0.85	0.14	0.62	0.20	0.48
Depth of lumbar Lordosis [mm] I	0.25	0.37	0.39	0.15	0.36	0.19	0.39	0.15	0.27	0.32	0.30	0.27	0.30	0.28	0.26	0.35
Lumbar angle [°] I	−0.19	0.50	−0.29	0.29	−0.27	0.32	−0.23	0.41	0.78	0.001 *	0.16	0.56	0.06	0.84	0.04	0.87
Inclination [°] II	−0.36	0.18	−0.06	0.82	−0.04	0.88	−0.06	0.83	−0.21	0.46	−0.04	0.88	−0.04	0.89	0.01	0.96
Depth of thoracic kyphosis [mm] II	−0.19	0.50	0.03	0.92	0.03	0.92	−0.03	0.91	−0.46	0.08	−0.14	0.60	−0.12	0.67	−0.10	0.71
Thoracic angle [°] II	0.34	0.21	−0.02	0.94	−0.02	0.94	0.02	0.95	0.38	0.15	0.62	0.01 *	0.58	0.02 *	0.61	0.02 *
Depth of lumbar Lordosis [mm] II	0.22	0.43	0.02	0.94	0.02	0.93	0.08	0.77	0.42	0.12	0.16	0.56	0.12	0.67	0.11	0.69
Lumbar angle [°] II	0.35	0.20	0.09	0.75	0.07	0.80	0.07	0.79	0.64	0.01 *	0.39	0.15	0.24	0.38	0.23	0.41
Inclination [°] III	−0.17	0.54	−0.36	0.18	−0.34	0.21	−0.34	0.21	−0.24	0.39	−0.06	0.82	−0.06	0.83	−0.02	0.94
Depth of thoracic kyphosis [mm] III	−0.19	0.50	−0.19	0.49	−0.18	0.52	−0.23	0.40	−0.41	0.12	−0.04	0.88	−0.03	0.92	−0.02	0.93
Thoracic angle [°] III	0.28	0.30	−0.14	0.61	−0.14	0.61	−0.07	0.80	0.30	0.27	0.54	0.04 *	0.51	0.05	0.51	0.05
Depth of lumbar Lordosis [mm] III	0.37	0.17	0.11	0.69	0.09	0.75	0.13	0.63	0.35	0.20	0.15	0.59	0.15	0.60	0.14	0.63
Lumbar angle [°] III	−0.14	0.63	−0.32	0.23	−0.31	0.26	−0.27	0.33	0.79	0.001 *	0.16	0.56	0.10	0.72	0.11	0.68

* Statistically significant correlation (*p* < 0.05).

**Table 4:** Results of Pearson correlations of posture parameters and LBP (VAS).

Posture Parameter	Group 1						Group 2					
	VAS I		VAS II		VAS III		VAS I		VAS II		VAS III	
	r	*p*	r	*p*	*r*	*p*	r	*p*	r	*p*	*r*	*p*
Inclination [°] I	0.37	0.17	0.09	0.76	0.17	0.54	−0.57	0.03 *	0.57	0.03 *	0.27	0.33
Depth of thoracic kyphosis [mm] I	0.35	0.20	0.14	0.61	0.32	0.24	−0.38	0.15	0.71	0.003 *	0.34	0.21
Thoracic angle [°] I	0.01	0.96	−0.34	0.20	−0.42	0.12	−0.19	0.48	−0.29	0.29	−0.05	0.86
Depth of lumbar Lordosis [mm] I	−0.32	0.24	−0.15	0.58	−0.31	0.26	0.41	0.13	−0.70	0.004 *	−0.34	0.20
Lumbar angle [°] I	0.19	0.49	0.09	0.74	−0.13	0.64	0.01	0.96	−0.56	0.03 *	−0.39	0.15
Inclination [°] II	−0.23	0.41	0.18	0.53	−0.09	0.75	−0.37	0.18	0.72	0.002 *	0.47	0.07
Depth of thoracic kyphosis [mm] II	−0.18	0.51	0.33	0.23	0.05	0.86	−0.14	0.62	0.78	0.001 *	0.46	0.08
Thoracic angle [°] II	−0.24	0.39	−0.72	0.002 *	−0.57	0.03 *	0.03	0.90	−0.21	0.44	−0.04	0.88
Depth of lumbar Lordosis [mm] II	0.18	0.51	−0.30	0.27	−0.03	0.90	0.09	0.74	−0.77	0.001 *	−0.49	0.06
Lumbar angle [°] II	−0.06	0.82	0.16	0.56	−0.29	0.30	−0.03	0.90	−0.44	0.10	−0.59	0.02 *
Inclination [°] III	0.26	0.34	0.30	0.28	0.15	0.60	−0.36	0.19	0.69	0.005 *	0.44	0.09
Depth of thoracic kyphosis [mm] III	0.28	0.31	0.50	0.05	0.46	0.08	−0.14	0.62	0.78	0.001 *	0.50	0.05
Thoracic angle [°] III	−0.11	0.70	−0.62	0.01 *	−0.59	0.02 *	0.11	0.68	−0.22	0.43	0.18	0.52
Depth of lumbar Lordosis [mm] III	−0.17	0.53	−0.48	0.07	−0.45	0.09	0.23	0.40	−0.76	0.001 *	−0.49	0.06
Lumbar angle [°] III	0.30	0.28	−0.01	0.97	−0.29	0.29	−0.03	0.92	−0.53	0.04 *	−0.64	0.01 *

* Statistically significant correlation (*p* < 0.05).

## Data Availability

The data presented in this study are available from the author upon request.
